# Temperature Modulates the Secretome of the Phytopathogenic Fungus *Lasiodiplodia theobromae*

**DOI:** 10.3389/fpls.2016.01096

**Published:** 2016-08-03

**Authors:** Carina Félix, Ana S. Duarte, Rui Vitorino, Ana C. L. Guerreiro, Pedro Domingues, António C. M. Correia, Artur Alves, Ana C. Esteves

**Affiliations:** ^1^Department of Biology, Centre for Environmental and Marine Studies, University of AveiroAveiro, Portugal; ^2^Department of Medical Sciences, Institute for Research in Biomedicine, University of AveiroAveiro, Portugal; ^3^Department of Physiology and Cardiothoracic Surgery, Faculty of Medicine, University of PortoPorto, Portugal; ^4^Department of Chemistry and QOPNA, University of AveiroAveiro, Portugal

**Keywords:** phytopathogenic fungi, extracellular enzymes, secretome, cytotoxicity, global changes

## Abstract

Environmental alterations modulate host–microorganism interactions. Little is known about how climate changes can trigger pathogenic features on symbiont or mutualistic microorganisms. Current climate models predict increased environmental temperatures. The exposing of phytopathogens to these changing conditions can have particularly relevant consequences for economically important species and for humans. The impact on pathogen/host interaction and the shift on their biogeographical range can induce different levels of virulence in new hosts, allowing massive losses in agricultural and health fields. *Lasiodiplodia theobromae* is a phytopathogenic fungus responsible for a number of diseases in various plants. It has also been described as an opportunist pathogen in humans, causing infections with different levels of severity. *L. theobromae* has a high capacity of adaptation to different environments, such as woody plants, moist argillaceous soils, or even humans, being able to grow and infect hosts in a wide range of temperatures (9–39°C). Nonetheless, the effect of an increase of temperature, as predicted in climate change models, on *L. theobromae* is unknown. Here we explore the effect of temperature on two strains of *L. theobromae* – an environmental strain, CAA019, and a clinical strain, CBS339.90. We show that both strains are cytotoxic to mammalian cells but while the environmental strain is cytotoxic mainly at 25°C, the clinical strain is cytotoxic mainly at 30 and 37°C. Extracellular gelatinolytic, xylanolytic, amylolytic, and cellulolytic activities at 25 and 37°C were characterized by zymography and the secretome of both strains grown at 25, 30, and 37°C were characterized by electrophoresis and by Orbitrap LC-MS/MS. More than 75% of the proteins were identified, mostly enzymes (glycosyl hydrolases and proteases). The strains showed different protein profiles, which were affected by growth temperature. Also, strain specific proteins were identified, such as a putative f5/8 type c domain protein – known for being involved in pathogenesis – by strain CAA019 and a putative tripeptidyl-peptidase 1 protein, by strain CBS339.90. We showed that temperature modulates the secretome of *L. theobromae*. This modulation may be associated with host-specificity requirements. We show that the study of abiotic factors, such as temperature, is crucial to understand host/pathogen interactions and its impact on disease.

## Introduction

It is widely accepted that the climate is changing at a global level. We will witness increased temperature and climatic extremes such as drought, floods, and storms (Piñeiro et al., 2010; Galant et al., 2012). Nonetheless, little effort has been directed to the identification of the impact that these forecasted conditions, specifically increased temperature, will have on microbial pathogen (MP)/host interactions (Eastburn et al., 2011; Gallana et al., 2013). Stress induced by increased temperature experienced by MPs will certainly impact the dynamics of host/pathogen interactions and ultimately result in changes in virulence (Lindner et al., 2010). Altered environmental conditions are causing many organisms to shift their biogeographic distribution ranges (MacDonald et al., 2008), and the same may be occurring with microorganisms (Azevedo et al., 2012; Bebber et al., 2013). The study of increased environmental temperature on the behavior of phytopathogens is therefore of extreme relevance.

Fungi can establish commensal and pathogenic relationships with their hosts that can be altered by discrete environmental changes, inducing a commensal relationship to evolve into a pathogenic one (Bliska and Casadevall, 2009). Furthermore, endophytes and plant pathogens have extraordinarily similar methods of invasion, suggesting a similarity of attributes related to the adaptation of a fungus to its host (van der Does and Rep, 2007; Hube, 2009; Blauth de Lima et al., 2016). A number of fungal molecules, like cell wall degrading enzymes (CWDEs), inhibitory proteins and enzymes involved in toxin synthesis, are known to contribute to fungal pathogenicity and virulence (King et al., 2011; Gonzalez-Fernandez and Jorrin-Novo, 2012). Proteomics is a powerful tool to identify unknown mechanisms underlying environmental alterations (Lemos et al., 2010; Alves et al., 2015). In this context, the analysis of the extracellular proteome, the secretome, allows identifying which proteins are involved in the interaction with the host and attempt to relate them with fitness and/or pathogenicity mechanisms (Bregar et al., 2012; Gonzalez-Fernandez and Jorrin-Novo, 2012; Gonzalez-Fernandez et al., 2015). The analysis of the secretome has been successfully made for phytopathogenic fungi, such as *Botrytis cinerea* (Zhang et al., 2014), *Diplodia corticola* (Fernandes et al., 2014) or *Verticillium albo-atrum* (Mandelc and Javornik, 2015).

*Lasiodiplodia theobromae* (Pat.) Griff. & Maubl. is a phytopathogenic fungus typical of the tropics and subtropics (Alves et al., 2008; Phillips et al., 2013). Despite being able to grow between 9 and 39°C, its optimal growth temperature is 27–33°C (D’souza and Ramesh, 2002). Widely distributed, it is mostly confined to 40° North and 40° South of the equator. Although *L. theobromae* has the ability to colonize healthy tissues without causing any harm (Jami et al., 2013), disease may appear if the plant is under stress. Therefore, it has been considered as a latent pathogen, capable of inducing endophytic infections (Jami et al., 2013). It has been associated to approximately 500 hosts, mostly woody plants, such as *Eucalyptus* spp. and to different fruit trees, like grapevines (Phillips et al., 2013; Rodríguez-Gálvez et al., 2015). *Lasiodiplodia theobromae* has also been associated to a number of cases of human infections, behaving as an opportunist (Summerbell et al., 2004; Kindo et al., 2010; Saha et al., 2012a,b). The most common cases are ocular infections but human death has been reported (Woo et al., 2008).

In this study the effect of temperature on two strains of *L. theobromae* was investigated; an environmental (CAA019) and a clinical strain (CBS339.90). *Lasiodiplodia theobromae* metabolome has been widely studied, but the enzymes, and other proteins, expressed by this organism have never been investigated. Therefore, the effect of temperature on the production of extracellular enzymes, on the secretome and on cytotoxicity of the secretome was evaluated.

## Materials and Methods

### Microorganisms

The strains used in this study were: CAA019, isolated from *Cocos nucifera* L. in Brazil, and CBS339.90, isolated from a phaeohyphomycotic cyst of patient from Jamaica (Alves et al., 2008). CBS339.90 was obtained from the Centraalbureau voor Schimmelcultures (CBS) Fungal Biodiversity Centre. Cultures were maintained on PDA (19.5 g.L^-1^; Potato Dextrose Agar; Difco).

### Radial Growth

Fungal growth was evaluated based on the development of the mycelium in solid media (PDA, Czapek, Oat Meal Agar and Corn Meal Agar). The plates were inoculated with a 7 mm-diameter agar plug from an actively growing fungal culture in PDA at 1 cm from the border of the plate and incubated at 25, 30, and 37°C. After 48 h, the colony radius was measured. Assays were carried out in triplicate and data is presented as average ± standard error.

### Biomass

Two plugs of 7 mm-diameter from an actively growing culture on PDA were inoculated on 50 mL of Potato Dextrose Broth (PDB) medium and incubated at 25, 30, or 37°C. After 24, 48, 72, 96, 120, 168, and 360 h (1–15 days), the mycelium was separated from the culture medium by filtration (filter paper). The mycelium was dried at 50°C for 48 h and the dry weight determined.

### Extracellular Enzymes

The different agar media plates were inoculated with a 7 mm-diameter agar plug from an actively growing culture and incubated at 25, 30, and 37°C for 48 h, unless otherwise stated. All assays were carried out in triplicate and data is presented as average ± standard error.

#### Detection and Quantification of Enzymatic Activity

The presence of caseinases, cellulases, amylases, xylanases, pectinases, ureases, and laccases was detected as described earlier (Esteves et al., 2014). Briefly, the various substrates [1% (w/v) skimmed milk, 0.5% (w/v) carboxymethylcellulose, 0.2% (w/v) starch, 0.5% (w/v) xylan, 0.5% (w/v) pectin, 2% (w/v) urea, and 1% (w/v) tannic acid, respectively] were independently added to a solution of 0.5% (w/v) malt extract and 1.5% (w/v) agar. The activities were detected by the formation of a halo around the mycelium (caseinases, ureases, and laccases) or after the addition of Lugol solution (amylases), Congo Red (cellulases and xylanases) or cetyltrimethyl ammonium bromide (pectinases).

Gelatinases were detected using a gelatin medium [1% (w/v) gelatin, 0.5% (w/v) malt extract, 1.5% (w/v) agar]. The plates were inoculated and the degradation of gelatin was detected as a clear halo around colonies, against an opaque background.

The activity was determined as a percentage of the maximum halo (cm) measured for each activity assayed.

#### Characterisation of Extracellular Enzymes by Zymography

Strains were grown as follows: two plugs of 7 mm-diameter from an actively growing culture on Potato Dextrose Agar (PDA) were inoculated on 50 mL of Potato Dextrose Broth (PDB) medium and incubated at the appropriate temperature for 28 days. Aliquots were taken every 48 or 72 h and stored at -80°C until analysis. The mycelium was separated from the culture medium by filtration (filter paper).

The characterisation of extracellular enzymes was accessed by zymography (Esteves et al., 2014). Extracellular media were diluted in sample buffer [2:1 (v/v); 62.5 mM Tris, pH 6.8, 10% SDS (w/v) and 20% glycerol (v/v)] and incubated at room temperature during 10 min. Proteins were then separated in lab-cast gels (10% polyacrylamide with the appropriated substrate) in a Mini-PROTEAN 3 (Bio-Rad) according to (Laemmli, 1970). Electrophoresis proceeded at 120 volts for 120 min at 4°C. After electrophoresis, the gel was washed twice with 0.25% Triton^®^ X-100 (v/v) for 60 min to remove SDS.

Gel analysis was performed after staining the proteins and scanned on a GS-800 Calibrated Densitometer (Bio-Rad). Quantity One v. 4.6.9 (Bio-Rad) was used to estimate the molecular mass of proteins and their optical densities. The apparent molecular weight (MW) of the proteins was determined using a MW calibration kit as marker, consisting of a mixture of proteins with 250, 150, 100, 75, 50, 37, 25, 20, 15, and 10 kDa (Precision Plus Protein Standard, Bio-Rad). Only gels where activity was detected are shown.

##### Xylanases

Xylanolytic activity was characterized by zymography, as described previously (Peterson et al., 2011) with slight modifications. One percent xylan was incorporated in the gel. After electrophoresis, the gel was incubated overnight at 25°C in 0.05 M Tris-HCl, pH 5.0, stained with Congo Red solution (1%) for 10 min. The gel was rinsed with a solution of 1 M NaCl. Enzymes with xylanolytic activity were detected as clear bands against a red background of non-degraded substrate.

##### Cellulases

Cellulolytic activity was assessed by zymography, as described previously (Peterson et al., 2011) with slight modifications. Carboxymethylcellulose (1%) was incorporated in the gel. After electrophoresis, the gel was incubated overnight at 25°C in 0.05 M Tris-HCl, pH 5, stained with Congo Red solution (1%) for 10 min. The gel was rinsed with 1 M NaCl. Enzymes with cellulolytic activity were detected as clear bands against a red background of non-degraded substrate.

##### Amylases

Amylolytic activity was assessed by zymography using 1% (w/v) starch, as described previously (Peterson et al., 2011) with slight modifications. After electrophoresis, the gel was incubated 3 h at 40°C in 0.05 M Tris-HCl, pH 5, stained with 1 mL Lugol’s iodine stock solution [(0.05 g I_2_, 0.1 g.mL^-1^ KI (Potassium Iodide)] in 50 mL distilled water. The gel was rinsed with distilled water. Enzymes with amylolytic activity were detected as clear bands against a dark background of non-degraded substrate.

##### Proteases

Gelatinolytic and caseinolytic activity was assessed by zymography, using 1% (w/v) gelatine or 1% (w/v) casein, as described previously (Duarte et al., 2009; Esteves et al., 2014), with slight modifications. After electrophoresis, the gel was incubated overnight, at room temperature, in 1.5 mM Tris, pH 8.8, 1 M NaCl, 1 M CaCl_2_, 2 mM ZnCl_2_, pH 7.4, stained with Coomassie Brilliant Blue R-250 [(in 50% ethanol (v/v), 10% acetic acid (v/v)] and destained with 25% ethanol (v/v), 5% acetic acid (bgv/v). Enzymes with gelatinolytic/caseinolytic activity were detected as clear bands against a blue background of non-degraded substrate.

### Protein Quantification

Protein quantification was made using BCA Protein Assay Kit (Pierce^TM^, Rockford, IL, USA), according to the manufacturer’s instructions. All the samples were quantified in triplicate.

### Secretome Analysis

Two mycelial plugs with 7 mm were used to inoculate the fungi into 50 mL of PDB medium. Cultures were grown for 72 h at 25, 30, and 37°C, in 250 mL Erlenmeyers flasks.

Extracellular medium of each strain was diluted (1:1) in loading buffer [2% (v/v) 2-mercaptoethanol, 2% (w/v) SDS, 8 M Urea, 100 mM Tris, 100 mM Bicine and traces of Bromophenol blue] and analyzed by electrophoresis (Laemmli, 1970). Lab-cast SDS-PAGE gels ran at 120 V for 2 h on 15% (w/v) acrylamide running gels. The running buffer contained 100 mM Tris, 100 mM Bicine and 0.1% (w/v) SDS. The samples were denatured at 100°C for 5 min prior to electrophoresis. Gel staining and image acquisition and analysis was as described before (Santos et al., 2013; Costa et al., 2014; Alves et al., 2015). All visible bands were manually excised and proteins were identified by Orbitrap LC mass spectrometry.

A Permutational Multivariate Analysis of Variance (RStudio) was employed using R package ‘vegan’ and the RStudio v 0.98.1103 interface (Oksanen et al., 2015; R Core Team, 2015) to understand which factor (strain or temperature) – if any – has the main effect on the protein profile of *L. theobromae*.

### Tryptic Digestion, Mass Spectrometry Analysis, and Protein Identification

Tryptic digestion was performed according to (Carvalhais et al., 2015), with a few modifications. Protein bands were manually excised from the gel and transferred to eppendorf tubes. Replicate bands were excised and also identified. The gel pieces were washed three times with 25 mM ammonium bicarbonate/50% acetonitrile (ACN, VWR Chemicals) and one time with ACN. The protein’s cysteine residues were reduced with 6.5 mM DTT and alkylated with 54 mM iodo-acetamide. Gel pieces were dried in a SpeedVac (Thermo Savant) and rehydrated in digestion buffer containing 12.5 μg.mL^-1^ sequence grade modified porcine trypsin (Promega) in 25 mM ammonium bicarbonate. After 90 min, the supernatant was removed and discarded, 100 μL of 25 mM ammonium bicarbonate were added and the samples were incubated overnight at 37°C. Extraction of tryptic peptides was performed by the addition of 10% formic acid (FA, Fluka)/50% ACN three times and finally with ACN. Tryptic peptides were lyophilized in a SpeedVac (Thermo Savant) and resuspended in 5% ACN/0.1% FA solution. The samples were analyzed with a QExactive Orbitrap (Thermo Fisher Scientific, Bremen) that was coupled to an Ultimate 3000 (Dionex, Sunnyvale, CA, USA) HPLC (high-pressure liquid chromatography) system. Prior to sample analysis, a complex mixture of peptides was obtained from the reduction, alkylation and tryptic digestion of six proteins (Sciex iTRAQ standard mixture), namely bovine serum albumin (P02769), *Escherichia coli* β-galactosidase (P00722), bovine α-lactalbumin (P00711), bovine β-lactoglobulin (P02754), chicken lysozyme C (P00698) and human serotransferrin (P02787). This peptide mixture was routinely used to test the nanoLC-MS/MS system performance, showing a protein identification coverage between 70 and 80% for a 100 ng injection.

The trap (5 mm × 300 μm I.D.) and analytical (150 mm × 75 μm I.D.) columns used were C18 Pepmap100 (Dionex, LC Packings), the latter having a particle size of 3 μm. Peptides were trapped at 30 μL.min^-1^ in 95% solvent A (0.1% FA/5% ACN v/v). Elution was achieved with the solvent B (0.1% formic acid/100% acetonitrile v/v) at 300 nL.min^-1^. The 50 min gradient used was as follows: 0–3 min, 95% solvent A; 3–35 min, 5–45% solvent B; 35–38 min, 45–80% solvent B; 38–39 min, 80% solvent B; 39–40 min, 20–95% solvent A; 40–50 min, 95% solvent A. Nanospray was achieved using an uncoated fused silica emitter (New Objective, Cambridge, MA, USA; o.d. 360 μm; i.d. 50 μm, tip i.d. 15 μm) biased to 1.8 kV. The mass spectrometer was operated in the data dependent acquisition mode. A MS2 method was used with a FT survey scan from 375 to 1600 m/z (resolution 35,000; AGC target 3E6). The 10 most intense peaks were subjected to HCD fragmentation (resolution 17,500; AGC target 5E4, NCE 25%, max. injection time 120 ms, dynamic exclusion 35 s). Spectra were processed and analyzed using Proteome Discoverer (version 2.0, Thermo), with the MS Amanda search engine (version 2.1.4.3751, University of Applied Sciences Upper Austria, Research Institute of Molecular Pathology). Uniprot (TrEMBL and Swiss-Prot) protein sequence database (version of May 2016) was used for all searches under *Macrophomina phaseolina*, *Neofusicoccum parvum*, *Botryosphaeria dothidea*, and *L. theobromae*. Database search parameters were as follows: carbamidomethylation and carboxymethyl of cysteine as a variable modification as well as oxidation of methionine, and the allowance for up to two missed tryptic cleavages. The peptide mass tolerance was 10 ppm and fragment ion mass tolerance was 0.05 Da. To achieve a 1% false discovery rate, the Percolator (version 2.0, Thermo) node was implemented for a decoy database search strategy and peptides were filtered for high confidence and a minimum length of six amino acids, and proteins were filtered for a minimum number of peptide sequences of 2 and only rank 1 peptides.

The subcellular localization of the identified proteins was deduced using Bacello (Pierleoni et al., 2006), as described before for Botryosphaeriaceae fungi (Fernandes et al., 2014) and function was obtained from Uniprot records.

### Cytotoxicity Assay

*In vitro* cytotoxicity evaluation was performed as described earlier (Cruz et al., 2013; Duarte et al., 2015) with slight modifications. Each strain was grown in PDB medium at 25, 30, and 37°C for 72, 96, and 120 h. The supernatants were filtered (0.20 μm pore size filter, Orange Scientific) and used to assess cytotoxicity. A Vero cell line (ECACC 88020401, African Green Monkey Kidney cells, GMK clone) was grown and maintained according to Ammerman et al. (2009). The microtiter plates were incubated at 37°C in 5% CO_2_ for 24 h. After cell treatment, the medium was removed by aspiration and 50 μL of DMEM with 10% resazurin (0.1 mg.mL^-1^ in PBS) was directly added to each well. The microtiter plates were incubated at 37°C in 5% CO_2_ until reduction of resazurin (Al-Nasiry et al., 2007). The absorbance was read at 570 and 600 nm wavelength in a microtiter plate spectrophotometer (Thermo scientific, Multiskan Spectrum).

## Results and Discussion

### Radial Growth and Biomass

Both strains were unable to grow at 5 and at 40°C and showed maximum radial growth at 30°C on PDA (considered the best growth conditions for these strains from this point forward). Czapek medium was the least adequate to the growth of *L. theobromae* (**Supplementary Figure S1**).

**FIGURE 1:**
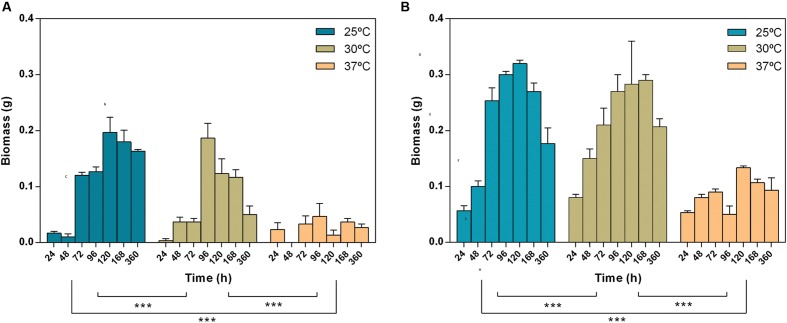
**Effect of time and temperature on the biomass of *Lasiodiplodia theobromae* [CAA019 **(A)** and CBS339.90 **(B)**].** Data is presented as average ± standard error. Two-way ANOVA (^∗∗∗^*p* < 0.001) was used to determine the statistical significance between the strains.

*Lasiodiplodia theobromae* biomass was determined (growth in liquid media; **Figure 1**). Both strains exhibit a biomass increase until 4 or 5 days of incubation; after this period, both strains start to degenerate with the consequent loss of biomass. The maximum biomass was obtained at 25°C. The biomass growth profile at 25, 30, and 37°C were significantly different (**Figure 1**, two-way Anova, *p* < 0.001). Strain CBS339.90 exhibited a similar growth pattern when compared with CAA019, with higher growth rates at 25 and 30°C although its biomass values were significantly higher (two-way Anova, *p* < 0.001) than those of the CAA019.

### Extracellular Enzymatic Activity

The extracellular enzymatic activity of *L. theobromae* was detected and quantified by plate assay and by zymography. Several extracellular enzymatic activities were tested at 25, 30, and 37°C by plate assay. The activities assayed are involved in the degradation of plant cell walls (as is the case of cellulases, xylanases, laccases, and pectinases), in the degradation of plant defenses (proteases) and in animal pathogenesis (gelatinases and ureases). Positive activities were evaluated by zymography.

**FIGURE 2:**
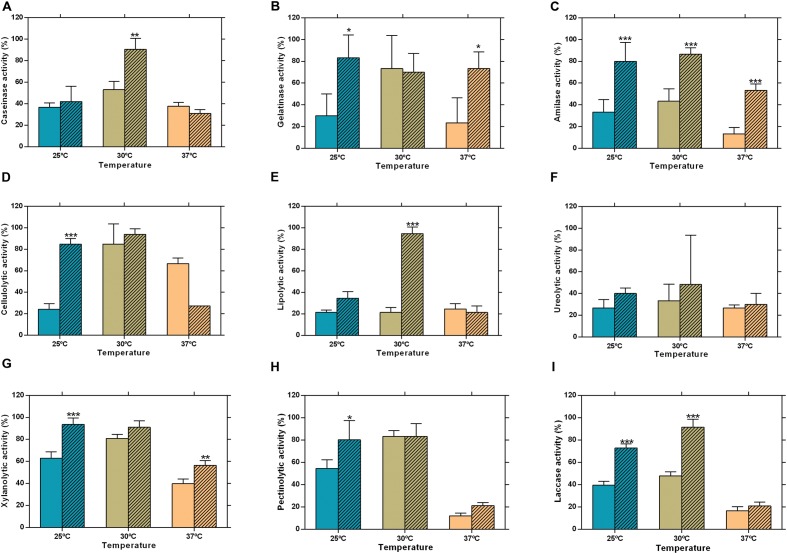
**Extracellular enzymatic activity of *L. theobromae* strains CAA019 (solid bars) and CBS339.90 (pattern bars) after a 48 h incubation period.** **(A)** caseinolytic, **(B)** gelatinolytic, **(C)** amylolytic, **(D)** cellulolytic, **(E)** lipolytic, **(F)** ureolytic, **(G)** xylanolytic, **(H)** pectinolytic, and **(I)** laccase activities. Data is presented as average ± standard error. Two-way ANOVA, followed by a Bonferroni multiple comparison test, was used to determine the statistical significance of extracellular enzymatic activity within the same temperature (^∗^*p* <0.05, ^∗∗^*p* < 0.01, ^∗∗∗^*p* < 0.001).

Due to the nature of the hosts – *C. nucifera* for CAA019 and a human patient for CBS339.90 – the zymographies were performed at 25 and 37°C, to investigate the effect that human body temperature could have on the strains. Both strains were able to secrete all enzymes assayed (**Figure 2**). Nonetheless, CBS339.90 displayed higher enzymatic activity than strain CAA019 in most conditions tested (**Figure 2**). Only one exception was detected; at 37°C CAA019 had a higher cellulolytic activity than CBS339.90 grown at the same temperature (*p* < 0.001; **Figure 2D**). Zymography analysis confirms the data obtained by plate assay. One exception was the cellulolytic activity of *L. theobromae*, which was higher at 25°C when analyzed by zymography, but not by plate assay. The difference observed could be related to the short culture time of *L. theobromae* in the plate assays.

**FIGURE 3:**
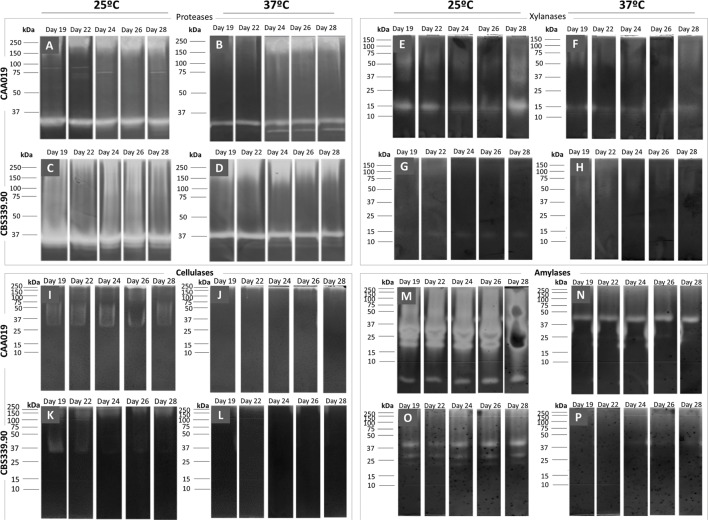
**Extracellular gelatinolytic **(A–D)**, xylanolytic **(E–H)**, amylolytic **(I–L)** and cellulolytic **(M–P)** activities of *L. theobromae*.** Strain CAA019 **(A/B, E/F, I/J, M/N)** and strain CBS339.90 **(C/D, G/H, K/L, O/P)** were grown for 28 days at 25°C **(A/C, E/G, I/K, M/O)** or 37°C **(B/D, F/H, J/L, N/P)**. Each gel is representative of three independent runs.

As seen in **Figures 2** and **3,** CAA019 and CBS339.90 express extracellular proteolytic, amylolytic, cellulolytic and xylanolytic enzymes. Most enzymes have very high (≈200 kDa) or very low (≈4.2 kDa) apparent MWs. These MWs could correspond to aggregation or multimeric forms of these enzymes or to degraded peptides with enzyme activity, rather than the MW of the discrete enzymes. In fact, the MWs of the enzymes identified by mass spectrometry (**Table 1**) are in the range of 26.9–58.7 kDa. We have observed a strain-, temperature-, and time-dependent expression pattern of many of these enzymes, particularly cellulases (**Figure 3**-M/N and O/P).

**Table 1:** Protein identification by LC-MS/MS of proteins secreted by *Lasiodiplodia theobromae* strains CAA019 and CBS339.90.

Bands^(1)^	Accession number	Proteins	Theoretical Mw (kDa)	Organism	Function^(2)^	Biological process^(2)^	Number of peptides^(3)^	Score MS AMANDA
1, 40	K2RRJ6	Glucose-methanol-choline oxidoreductase	72	*Macrophomina phaseolina*	Oxidoreductase activity, acting on the CH-OH group of donors, other acceptors	Catalysis of an oxidation-reduction reaction	2	679.27
1, 40	R1E7Q5	Putative choline dehydrogenase protein	71.3	*Neofusicoccum parvum*	Oxidoreductase activity, acting on the CH-OH group of donors, other acceptors	Catalysis of an oxidation-reduction reaction	2	679.27
7	R1GTC8	Putative tripeptidyl-peptidase 1 protein	64.8	*Neofusicoccum parvum*	Serine-type endopeptidase activity	Proteolysis	2	426.40
8	K2RUW5	Phosphoesterase	43.9	*Macrophomina phaseolina*	Hydrolase activity, acting on ester bonds	Hydrolase	2	663.55
9	R1GU94	Putative glucan endo–α-glucosidase agn1 protein	49.3	*Neofusicoccum parvum*	Hydrolase activity	Hydrolase	2	579.98
10	K2RGL3	Peptidase A1	26.9	*Macrophomina phaseolina*	Aspartic-type endopeptidase activity	Proteolysis	2	4710.33
6, 11	K2SBN0	β-xylanase	34.2	*Macrophomina phaseolina*	Hydrolase activity	Carbohydrate metabolic process, metabolic process	2	1020.33
6, 11	R1FWZ0	β-xylanase	34.8	*Neofusicoccum parvum*	Hydrolase activity	Carbohydrate metabolic process, metabolic process	2	1020.33
16	K2SSA3	β-galactosidase	107.8	*Macrophomina phaseolina*	β-galactosidase activity	Carbohydrate metabolic process	2	382.31
18	R1GH64	Putative f5 8 type c domain protein	59.2	*Neofusicoccum parvum*	Hydrolase activity, hydrolyzing *O*-glycosyl compounds	Carbohydrate metabolic process, cell adhesion	2	2398.24
19, 20, 41	K2RQR5	Peptidase A1 (Fragment)	39.9	*Macrophomina phaseolina*	Aspartic-type endopeptidase activity	Proteolysis	2	781.41
5, 10, 13, 15,21, 24, 30, 31, 32, 33, 37, 42, 44	R1ESA5	Putative a chain endothiapepsin	42.5	*Neofusicoccum parvum*	Aspartic-type endopeptídase activity	Proteolysis	2	5116.87
7, 25	K2S7L9	Glucoamylase	67.8	*Macrophomina phaseolina*	Glucan 1,4-α-glucosidase activity; starch binding	Polysaccharide catabolic process	2	5006.77
9, 28, 36	K2R498	Glycoside hydrolase family 71	49.2	*Macrophomina phaseolina*	Hydrolase activity	Hydrolase	2	2124.51
6, 12, 22, 29, 43	K2STT8	Glycoside hydrolase family 17	32	*Macrophomina phaseolina*	Carbohydrate metabolic processes	Hydrolase activity	2	5379.70
1, 7, 25, 26, 27, 34,40	R1GLG1	Glucoamylase	68.5	*Neofusicoccum parvum*	Glucan 1,4-α-glucosidase activity; starch binding	Polysaccharide catabolic process	4	9860.40


**FIGURE 4:**
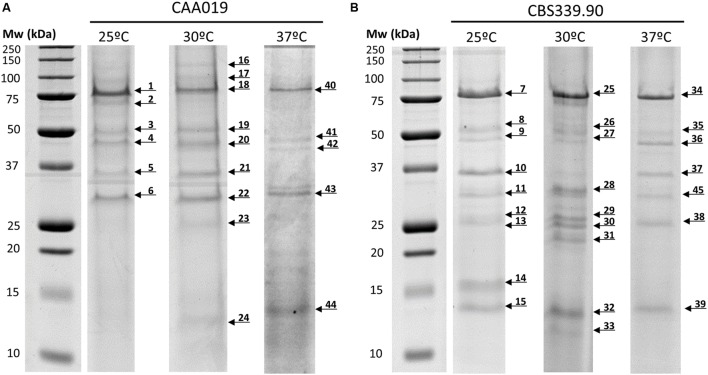
**Secretome of *L. theobromae* strains CAA019 **(A)** and CBS339.90 **(B)** analyzed by SDS-PAGE.** Cultures were grown for 72 h, at 25 and 37°C. Each gel is representative of three independent analyses. Arrows represent the different proteins found in these conditions.

*Band number corresponds to the numbering indicated in **Figure 4**^(1)^. The GO terms correspondent to the function and the biological processes^(2)^ of the proteins were searched with Interpro (Hunter et al., 2012). The subcellular localization was deduced with Bacello (Pierleoni et al., 2006): all proteins were inferred to be extracellular. Only proteins with two unique peptides identified were included in the table. The number of peptides^(3)^ is the number of peptides identified per band.*

Fungal pathogens have a detrimental impact on plant production and the strategies they use to infect their hosts should be investigated to predict their behavior and protect the plants from fungal infections (Gonzalez-Fernandez and Jorrin-Novo, 2012; Fernandes et al., 2014). Fungal pathogenicity results in the synthesis of molecules, such as CWDEs, inhibitory proteins and toxins, that have been described as being involved in the infection mechanisms of fungi (Kikot et al., 2009). We expect that a plant adapted pathogen will secrete enzymes able to degrade plant specific substrates (as is the case of CWDEs) while an animal adapted strain will secrete a lower number (or exhibit a lower activity) of these enzymes. On the other hand, we expect that a strain adapted to animal environment will express a higher number of enzymes able to interact with mammalian specific substrates.

Cellulose, hemicellulose, and lignin are the major components of the plant cell wall, cellulose being the most abundant (Gibson et al., 2011). The ability of fungi to produce CWDEs facilitate fungal penetration (Gibson et al., 2011). There are some evidences that plant pathogens may produce different amounts of specific CWDEs depending on the plant host (van der Does and Rep, 2007; King et al., 2011). In this context it is curious that both strains have such distinct cellulolytic activity profiles. It is plausible that strain CBS339.90 may have developed some type of adaptation to colonize human hosts. For example the high protein-content matrix (without complex carbohydrates like cellulose) may have contributed to the high secretion of proteases by CBS339.90.

### Secretome Analysis

The secretome of both strains of *L. theobromae* grown at 25, 30, and 37°C (**Figure 4**) was analyzed by SDS-PAGE/LC/MS/MS. Approximately 77% of the selected proteins were identified (10 proteins were identified for CAA019 strain and 11 for CBS339.90 strain); most of proteins are extracellular enzymes (87.5%) and only 12.5% are extracellular proteins with non-enzymatic functions (**Table 1** and **Supplementary Table S1**).

Due to the lack of genome sequence, all proteins were identified based on their homology with the proteins of *M. phaseolina* MS6 and *N. parvum* UCRNP2, two pathogenic members of the family Botryosphaeriaceae, whose genome is sequenced and integrated into UniProtKB (Islam et al., 2012).

The strains showed different protein profiles, which were affected by growth temperature, suggesting different interactions with the environment. For each strain some unique proteins were found.

For CAA019 we identified four strain-specific proteins – a glucose-methanol-choline oxidoreductase (K2RRJ6), a putative choline dehydrogenase protein (R1E7Q5), a β-galactosidase (K2SSA3) and a putative f5/8 type c domain protein (R1GH64). From these, the three enzymes are known to be involved in cellulose degradation (Yoshida et al., 2002; Van den Brink and de Vries, 2011), which is expected, since this is a phytopathogenic strain. However, the putative f5/8 type c domain protein, found only at 30°C, is a coagulation factor. The coagulation factor expressed by CAA019 possesses a functional domain that promotes binding to anionic phospholipids (Hunter et al., 2012), known to be involved in pathogenesis for some species of fungi (Chaffin et al., 1998).

For CBS339.90, also four strain-specific proteins were identified – a putative tripeptidyl-peptidase 1 protein (R1GTC8), a phosphoesterase (K2RUW5), a putative glucan endo-α-glucosidase agn1 protein (R1GU94) and a glycoside hydrolase family 71 (K2R498). It is important to highlight that the putative tripeptidyl-peptidase 1 protein was found only at 25°C. This protease has a serine-type endopeptidase activity (Hunter et al., 2012). In *Aspergillus fumigatus*, it is part of a set of proteases (sedolisins) that have the ability to degrade proteins at acidic pH values. This allows the generation of assimilable nitrogen in decomposing organic matter and composts (Reichard et al., 2006). Also, it is responsible to acidify the culture supernatant of this species *in vitro*, which can be related with the acidification of its microenvironment in the living host to facilitate nutrition and proliferation of the hyphae during the infection process (Reichard et al., 2006). The glycoside hydrolases are typically produced by phytopathogenic fungi to degrade cellulose and xylans of the plant cell wall and penetrate into the host tissue (Murphy et al., 2011). These enzymes act hydrolyzing the glycosidic bonds between two or more carbohydrates or between a carbohydrate and a non-carbohydrate moiety. The GH family 71 was expressed only by CBS339.90 and comprises the α-1,3-glucanases (Hunter et al., 2012).

Other families of GH were found in both strains, as the GH family 10, that includes xylanases and cellobiohydrolases and GH family 17, that comprises enzymes as endo-1,3-β-glucosidades, lichenases and exo-1,3-glucanases (Hunter et al., 2012). Glucoamylases whose function is also related with the degradation of plant cell wall, by hydrolyzing 1,4-α-glucose (Hunter et al., 2012; Kubicek et al., 2014) are also expressed by both strains.

Several aspartic proteases are expressed by *L. theobromae*. Aspartic proteases from the family A1 and a putative a chain endothiapepsin are expressed by both strains. Besides its involvement in physiologic cellular functions, aspartic proteases play a crucial role as virulence factors, dissemination, and host evasion (Rawlings and Bateman, 2009). These enzymes have been related to human pathogenesis (Monod et al., 2002; Yike, 2011), which is concordant with the fact that we identified these enzymes at 37°C. In these cases, aspartic proteases are probably involved in several processes such as the degradation of the extracellular matrix (mainly composed by collagens and other proteins; Duarte et al., 2007, 2016) leading to the progression of the pathogen.

Thus, some of the proteins seem to be involved in plant pathogenesis processes, as is the case of glycoside hydrolases (Murphy et al., 2011), but also in animal pathogenesis processes, as is the case of proteases or aspartic proteases. The plate assay confirmed the presence of these enzymes for both isolates and the zymography analysis, the presence of multiple endoglucanases, xylanases, and proteases (**Figure 3**).

Globally, both strains showed a similar tendency regarding the number of detected bands, with a decrease of the number with increasing temperature. However, CBS339.90 strain presented more detected bands when compared with CAA019 strain (**Figure 4**; **Table 2**).

**Table 2:** Summary of the proteins identified in *L. theobromae* strains CAA019 and CBS339.90 at 25, 30, and 37°C.

Accession number	Proteins	CAA019			CBS339.90		
			
		25°C	30°C	37°C	25°C	30°C	37°C
K2RRJ6	Glucose-methanol-choline oxidoreductase	+	-	+	-	-	-
R1E7Q5	Putative choline dehydrogenase protein	+	-	+	-	-	-
R1GTC8	Putative tripeptidyl-peptidase 1 protein	-	-	-	+	-	-
K2RUW5	Phosphoesterase	-	-	-	+	-	-
R1GU94	Putative glucan endo–α-glucosidase agn1 protein	-	-	-	+	-	-
K2RGL3	Peptidase A1	-	-	-	+	-	-
K2SBN0	β-xylanase	+	-	-	+	-	-
R1FWZ0	β-xylanase	+	-	-	+	-	-
K2SSA3	β-galactosidase	-	+	-	-	-	-
R1GH64	Putative f5 8 type c domain protein		+				
K2RQR5	Peptidase A1 (Fragment)		+	-			
R1ESA5	Putative a chain endothiapepsin	+	+	+	+	+	+
K2S7L9	Glucoamylase				+	+	
K2R498	Glycoside hydrolase family 71				+	+	+
K2STT8	Glycoside hydrolase family 17	+	+	+	+	+	
R1GLG1	Glucoamylase	+		+	+	+	+


*The symbols represent the presence (+) or the absence (-) of the proteins.*

A Permutational Multivariate Analysis of Variance was employed using R package ‘vegan’ and the RStudio v 0.98.1103 interface (Oksanen et al., 2015; R Core Team, 2015). It was shown that for the identified proteins (computed through a presence-or-absence matrix), 44% of the variance is explained by the strain factor (*R*^2^ = 0.4467). Contrary, the differences found for fungi growth temperature were not statistically significant, suggesting that the strain has a large relevance on the protein profile in this study (*p*-value < 0.01), which could be related to a host-adaptation of these strains.

### Cytotoxicity

**FIGURE 5:**
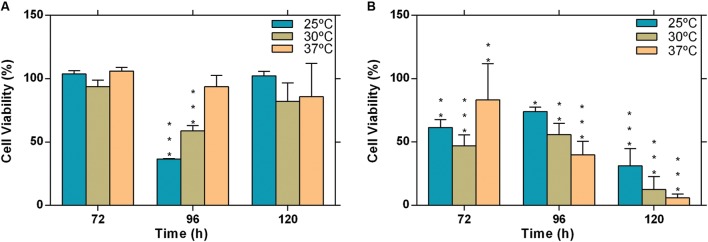
**Evaluation of cytotoxicity of the extracellular fraction of *L. theobromae*.** **(A)** strain CAA019 incubated at 25, 30, and 37°C and **(B)** strain CBS339.90, incubated at 25, 30, and 37°C. Data is presented as average ± standard error of two independent experiments performed in triplicate. Two-way ANOVA, followed by a Bonferroni multiple comparison test, was used to determine the statistical significance of cytotoxicity within the same temperature (^∗^*p* < 0.05, ^∗∗^*p* < 0.01, ^∗∗∗^*p* < 0.001).

We investigated the influence of temperature on the cytotoxic potential of *L. theobromae*, strains CAA019 and CBS339.90 to Vero cell line. Both strains were cytotoxic under all conditions tested, with the exception of the environmental strain (CAA019) grown at 37°C (**Figure 5**). Interestingly, temperature had opposite effect on both strains; the cytotoxic effect of CAA019 was detected mostly when cultured at 25°C, decreasing at 30°C and being absent at 37°C (**Figure 5A**). The cytotoxic effect of CBS339.90 (**Figure 5B**) increased with temperature (*p* < 0.001), reaching 90% of cell mortality when grown at 37°C. Interestingly, CBS339.90 also showed higher growth rates (**Figure 1**) and extracellular enzymatic activity (**Figure 2**) at 37°C when compared with CAA019. Proteins typically related with infection mechanisms were found in the secretome of CBS339.90 (aspartic proteases), suggesting that they can be involved in the high level of cell mortality found for this strain.

Host specificity in plant-pathogenic fungi impact their host range. Although it has been suggested that for most Botryosphaeriaceae species there is no host specificity (Esteves et al., 2014), the differences between the secretome profiles of both strains at 25 and 37°C can be related to adaptation to specific host conditions. While strain CAA019 was isolated from a coconut tree, CBS339.90 was isolated from a human, at approximately 37°C. Since the optimal growth of this species is between 27 and 33°C, we can argue that the ability to infect humans may be the result of an adaptation to an increased temperature. This agrees with the fact that only strain CBS339.90 was cytotoxic to Vero cells at 37°C and that it produces more biomass and extracellular enzymes at this temperature than CAA019.

## Conclusion

We showed that temperature modulates the extracellular protein production of strains of *L. theobromae* found in different ecological niches: a tropical fruit tree and a hospitalized patient. The temperature growth range was wide, between 15 and 37°C, a feature common for species in the family Botryosphaeriaceae. The extracellular enzymatic activity also varied with fungal growth temperature.

Both strains were able to induce cytotoxic effects in mammalian cells. However, CBS339.90 is more cytotoxic than CAA019, especially at 37°C, where cell mortality reached 90%.

The ability to grow at 37°C and the secretion of hydrolytic enzymes, namely of aspartic proteases, at this temperature are typical characteristics of human pathogenic fungi that may be related to virulence (Karkowska-Kuleta et al., 2009). Our data suggests that the colonization of different hosts may lead to strain specificity.

## Author Contributions

AA, AC, AE, AD, and PD conceived and designed the experiments. CF, AD, AG, and RV performed the experiments. AE, CF, AD, AG, and RV analyzed the data. AE, AD, and CF wrote the paper. AA, AC, AE, AD, and PD edited and approved the manuscript. All authors approved the final version of the manuscript.

## Conflict of Interest Statement

The authors declare that the research was conducted in the absence of any commercial or financial relationships that could be construed as a potential conflict of interest.

## Supplementary Material

**FIGURE S1 | Effect of temperature and culture medium on the growth of *Lasiodiplodia theobromae*.** Radial growth of *L. theobromae*, CAA019 **(A)** and CBS339.90 **(B)**, grown at different temperatures (between 5 and 40°C) and in different culture media was determined after 48 h of incubation. Data is presented as average ± standard error. Two-way ANOVA, Bonferroni test, was used to determine the statistical significance compared to 30°C (^∗^*p* < 0.05 and ^∗∗∗^*p* < 0.001, for OMA; ¥*p* < 0.05 and ¥¥¥*p* < 0.001, for CMA; $*p* < 0.05 and $$$*p* < 0.001, for PDA; #*p* < 0.05 and ###*p* < 0.001, for Czapek).

**TABLE S1 | List of identified proteins by Orbitrap LC-MS/MS using Proteome Discoverer 2.0 software.** The table indicates the protein accession number, the information on protein group master (a master protein is the representative of a group of homologous proteins), protein name, the sequence coverage achieved in percentage, number of peptides identified (at least two peptides), number of peptide-spectrum matches (PSM), number or unique peptides (for a certain species), number of protein groups to which the protein belongs, molecular weight (MW) in kDa, the matched peptide sequences obtained by MS/MS (Sequence) and the protein score as given by MS Amanda algorithm, respectively.
